# Leptin modulates the expression of catabolic genes in rat nucleus pulposus cells through the mitogen-activated protein kinase and Janus kinase 2/signal transducer and activator of transcription 3 pathways

**DOI:** 10.3892/mmr.2015.3646

**Published:** 2015-04-20

**Authors:** DAOYI MIAO, LINGZHOU ZHANG

**Affiliations:** Department of Orthopedic Surgery, The Third Affiliated Hospital of Wenzhou Medical University, Ruian, Zhejiang 325200, P.R. China

**Keywords:** leptin, nucleus pulposus, interleukin-1β, catabolism

## Abstract

Obesity has been demonstrated to be involved in the progress of intervertebral disc degeneration (IDD). However, the associated mechanisms remain to be elucidated. The purpose the present study was to examine the effect of leptin on the expression of degeneration-associated genes in rat nucleus pulposus (NP) cells, and determine the possible mechanism. Normal NP cells, obtained from Sprague Dawley rats, were identified using immunocytochemistry for the expression of collagen II and CA125, and treated with leptin and/or interleukin (IL)-β. Subsequently, the mRNA expression levels of matrix metalloproteinase (*MMP)-1*, *MMP-3*, *MMP-9*, *MMP-13*, a disintegrin and metalloproteinase with thrombospondin motifs *(ADAMTS)-4*, *ADAMTS-5*, *aggrecan* and *COL2A1* were detected by reverse transcription-quantitative polymerase chain reaction (RT-q-PCR). Alcian staining and immunocytochemistry were used to examine the expression levels of proteoglycan and collagen II. The pathway activation was investigated using western blotting, and inhibitors of the pathways were used to reveal the effect of these pathways on the NP cells. The results of the RT-qPCR demonstrated that leptin alone upregulated the mRNA expression levels of *MMP-1*, *MMP-13*, *ADAMTS-4*, *ADAMTS-5* and *COL2A1*. Synergy of leptin and IL-β was found in the increased expression levels of MMP-1, MMP-3 and ADAMTS-5. The leptin-treated NP cells exhibited decreased expression of collagen II. The mitrogen-activated protein kinase (MAPK) pathway (c-Jun-N-terminal kinase, phosphorylated extracellular signal-regulated kinase and p38), phosphatidylinositol 3-kinase (PI3K)/Akt pathway and Janus kinase (JAK)2/signal transducer and activator of transcription 3 pathway were all activated by leptin, however, inhibitors of all the pathways, with the exception of the PI3K/Akt pathway, reversed the expression levels of MMP-1 and MMP-13. These results suggested that leptin promoted catabolic metabolism in the rat NP cells via the MAPK and JAK2/STAT3 pathways, which may be the mechanism mediating the association between obesity and IDD.

## Introduction

There are several factors, particularly obesity, which lead to intervertebral disc degeneration (IDD), which is the predominant origin contributing to low back pain (LBP) (1,2). These factors may lead to the disturbance of balance between anabolism and catabolism in the extracellular matrix (ECM), including type II collagen, type I collagen and proteoglycan (predominantly aggrecan) (3,4). In the disc, proteoglycan and collagen II are expressed predominantly in the nucleus pulposus (NP). The balance of ECM metabolism is regulated by catabolic enzymes, including matrix metalloproteinases (MMPs), a disintegrin and metalloproteinase with thrombospondin motifs (ADAMTS) and their inhibitors, secreted by the intervertebral disc cells, which determine the progression of IDD (5). The catabolic enzymes, which degrade components of the ECM can disturb this balance and impair ECM turnover (5–7). In addition, the well-known pro-inflammatory mediator, interleukin (IL)-1β can induce disc degeneration, in part, via upregulation of the destructive enzymes (8). In the present study, IL-β was used in combination with leptin to confirm the effect of leptin on the levels of degeneration-associated genes.

Leptin, a 16kDa peptide hormone, is predominantly secreted by the adipose tissue, which regulates obesity and is one of the factors contributing to IDD (2). Leptin can regulate energy homeostasis, including food intake, and can also exert its effect as an endocrine hormone on immune function, bone formation and neuroendocrine function (9–12). Previously, the presence of leptin and its acceptors has been confirmed in human NP and annulus fibrosus (AF) cells in several studies (13,14). In addition, it has been suggested that leptin may be important in modulating the expression of catabolic enzymes in chondrocytes (15,16). Leptin, alone or in combination with IL-1β has been observed to increase the levels of MMP-1, MMP-3 and MMP-13 in human osteoarthritic cartilage and chondrocytes (15,16). Furthermore, leptin mediates the inflammatory activities of the chondrocytes, in which the expression levels of IL-1β, nitric oxide and IL-8 are elevated (17,18). To the best of our knowledge, the biochemical properties of NP cells are similar to those of chondrocytes, however, the effect of leptin on the catabolic enzymes within the NP cells remains to be fully elucidated. The present study hypothesized that leptin enhances the levels of catabolic genes and impairs the synthesis of proteoglycan and collagen II.

The leptin receptors, as products of the diabetes gene, are comprised of five forms, including long (OB-Rb), short (OB-Ra, -Rc, and -Rd), and soluble (OB-Re) receptors (18,19). However, the OB-Rb receptor, regarded as the predominant functional receptor, mediates the most biological effects of leptin with the assistance of the other forms within the body (20). In the disc and NP cells, the presence of OB-Rb and OB-Ra has been demonstrated (21). When binding with the receptors, leptin, considered pro-inflammatory and pro-catabolic factor in certain cells, activates several signaling pathways, including the MAPK (c-Jun-N-terminal kinase, extracellular signal-regulated kinase and P38) pathways, and the protein kinase C, phosphatidylinositol 3-kinase (PI3K)/Akt, Janus kinase (JAK)2/signal transducer and activator of transcription (STAT)3 and nuclear factor (NF)-κB pathways. Since the MAPK, JAK2/STAT3 and Akt pathways are present in chondrocytes (15,16), the present study examined whether these pathway were involved in gene expression in NP cells.

The present study aimed to determine whether leptin, alone or in combination with IL-β, regulates catabolic metabolism in NP cells using reverse transcription-quantitative polymerase chain reaction (RT-q-PCR) and western blotting. The present study then aimed to detect the mechanism by which leptin exerts its effect via the use of specific pathway inhibitors.

## Materials and methods

### Materials

The pathway inhibitors were all obtained from Merck Millipore (Nottingham, UK). IL-1β, recombinant rat leptin and Alcian blue were acquired from Sigma-Aldrich (St. Louis, MO, USA). The agents used for cell culture were all purchased from Gibco Life Technologies, Carslbad, CA, USA). The primary antibodies against collagen II and cytokeratin 19 were from Abcam (Cambridge, UK), and those against the protein of the signaling pathways were from Cell Signaling Technology, Inc. (Danvers, MA, USA). The primers used for RT-qPCR were designed and obtained from Invitrogen Life Technologies (Carslbad, CA, USA).

### NP cell culture and treatment

The experimental procedures involving the use of animals were approved by the Animal Care and Use Committee of Ruian People’s Hospital (Ruian, China). A total of 30 male Sprague-Dawley rats (3-month-old, 250–300 g; Shanghai Laboratory Animal Center, Shanghai, China) were sacrificed using 10% (w/v) chloral hydrate (3.5 ml/kg body weight; Sigma-Aldrich, St. Louis, MO, USA). The spinal columns between L1 and L6 were removed en bloc, and the NP tissue was detached using a dissecting microscope (Xintian Medical Device, Zhenjiang, China). When the cartilage endplate was cut open, the gelatinous nucleus pulposus extruded out the endplate under the relative high pressure in the discs. The tissue was then treated with 0.1% collagenase II for 4 h at 37°C, followed by filtration. The cells (5×10^6^ cells/25 cm^2^ flask) were cultured in Dulbecco’s modified Eagle’s medium (DMEM) with 10% fetal bovine serum (FBS) and antibiotics (1% penicillin/streptomycin) in an incubator in 5% CO_2_ at 37°C. At 80–90% confluence, the cells were harvested with 0.25% trypsin-EDTA (Gibco Life Technologies, Carslbad, CA, USA), counted and replanted into 10 cm culture plates at the appropriate density. The second-passage cells, cultured in a monolayer, were used in the subsequent experiments. All experiments were approved by the ethics committee of Wenzhou Medical University, Ruian People’s Hospital, Ruian, China.

The NP cells (1×10^6^ cells/well in 6-well plates) were incubated with DMEM and 2% FBS for 12 h prior to pretreatment with the indicated pathway inhibitors and IL-1β (10 ng/ml), which were added 30 min prior to the addition of recombinant rat leptin. For RT-qPCR, the NP cells were either treated with different concentrations of leptin (0.1, 1 or 10 *µ*g/ml), were co-treated with leptin (10 *µ*g/ml) and IL-1 β (10 ng/ml), or with leptin (10 *µ*g/ml) combined with the following corresponding pathway inhibitors: SP600125, a JNK inhibitor (10 *µ*M); U0216, a ERK inhibitor (5 *µ*M); SB220025, a p38 inhibitor (10 *µ*M); Wortannim, a PI3K/Akt inhibitor (20 nM) or AG490, a JAK2/STAT3 inhibitor (40 *µ*M) for 48 h. For Alcian staining and immunocytochemistry, the NP cells were incubated with leptin (10 *µ*g/ml) for 1 week. For western blotting, the cells were either treated with leptin (10 *µ*g/ml) alone for 10 and 60 min or co-treated with leptin and the corresponding inhibitor for 60 min at 37°C.

### Immunocytochemistry and immunofluorescence

The NP cells (1×10^6^ cells/well in 6-well plates) were fixed with fresh 4% paraformaldehyde (Sigma-Aldrich) for 10 min at 37°C in 24-well plates and rinsed with phosphate-buffered saline (PBS’ Sigma-Aldrich) three times. The cells were then treated with 0.2% Triton X-100 (Sigma-Aldrich) for 15 min. Following blocking with 5% goat serum (Gibco Life Technologies) for 30 min at 37°C, the cells were incubated with primary antibodies against collagen II (Rabbit anti-rat polyclonal; 1:100; cat. no. ab34712; Abcam), Cambridge, UK) and cytokeratin 19 (Rabbit anti-rat polyclonal; 1:100; cat. no. ab101255; Abcam) overnight. For immunocytochemistry, these cells were incubated with horseradish peroxidase (HRP)-conjugated secondary antibodies (Goat anti-rabbit monoclonal; 1:50; cat. no. A0208; Beyotime Institute of Biotechnology, Haimen, China) for 2 h at room temperature, followed by counterstaining with hematoxylin (Beyotime Institute of Biotechnology). For immunofluorescence, the cells were incubated with fluorescein isothiocyanate (FITC)-labeled secondary antibodies (Goat anti-rabbit monoclonal; 1:100; cat. no. A0562; Beyotime Institute of Biotechnology), followed by staining with 4′,6-diamidino-2-phenylindole (Beyotime Institute of Biotechnology) for nuclei. Finally, these cells were observed under light or fluorescence microscopy (Olympus, Tokyo, Japan).

### Alcian blue staining

To analyze the expression of proteoglycans, the fixed cells were stained with 1% Alcian blue solution for 30 min at 37°C, which was dissolved in 3% glacial acetic acid. The cells were rinsed with a tap water for 5 min and counterstained with 0.1% nuclear red solution dissolved in 5% aluminum sulfate for 20 min. The staining density was analyzed under light microscopy.

### Western blotting

The total protein was isolated using radioimmunoprecipitation assay lysis buffer with 1 mM phenylmethylsulfonyl fluoride (Beyotime Institute of Biotechnology), and the protein concentration was determined with using an Enhanced BCA Protein Assay kit (Beyotime Institute of Biotechnology,). Equal quantities (30 *µ*g) of protein from each sample were separated using sodium dodecyl sulfate-polyacrylamide gel electrophoresis and transferred onto polyvinylidene difluoride membranes (Bio-Rad Laboratories, Inc., Hercules, CA, USA). Following blocking with 5% nonfat milk, the membranes were incubated with the following rabbit anti-rat primary antibodies overnight: p-JNK (cat. no. 9251), JNK (cat. no. 9252). p-ERK (cat. no. 4370), ERK (cat. no. 9102), p-p38 (cat. no. 9211), p38 (cat. no. 8690), p-Akt (cat. no. 13038), Akt (cat. no. 9272) p-STAT3 (cat. no. 9145), STAT3 (cat. no. 4904) and GAPDH (cat. no. 5174), all purchased from Cell Signaling Technology, Inc. and diluted 1:1,000. Following this, the membranes were incubated with HRP-conjugated secondary antibodies (Goat anti-rabbit; 1:2,000; cat. no. A0208; Beyotime Institute of Biotechnology). All the antibodies were diluted to 1:1,000. Finally, the bands were detected using ECL plus reagent(Invitrogen Life Technologies) on an enhanced chemiluminescence detection system (PerkinElmer, Inc., Waltham, MA, USA). In addition, the intensity of the bands were quantified using Alpha Ease FC 4.0 software (Alpha Innotech Corp., San Leandro, CA, USA).

### RT-qPCR

The total RNA was extracted using TRIzol reagent (Invitrogen Life Technologies). The RNA (1 *µ*g) was used to synthesize cDNA (MBI Fermantas, St. Leon-Rot, Germany). For qPCR amplification, a 20 *µ*l reaction volume, containing 10 *µ*l 2X SYBR Premix Ex Taq mixture (Takara, Bio Inc., Otsu, Japan), 0.2 *µ*mol/l each primer, 2 *µ*l 2-fold diluted cDNA and sterile distilled water was used. The reaction and detection were performed using a light cycle (Roche, Mannheim, Germany). The PCR cycling steps were as follows: 96°C for 5 min; 35 cycles of 94°C for 30 sec, 60°C for 30 sec and 72°C for 60 sec; followed by 72°C for 5 min for extension. The primers used are shown in Table I. The cycle threshold (Ct) values were obtained and normalized to the housekeeping gene, glyceraldehyde phosphate dehydrogenase. When the fluorescence intensity reached 0.05, the Ct value was determined as the cycle number and following confirmation that in this range all curves were in the exponential phase of amplification, the value was selected. The ΔΔCt method was used to calculate the relative mRNA levels of each target gene. ΔΔCt method: ΔCt = Ct_target_ - Ct_GAPDH_, ΔΔCt = ΔCt_treated_ - ΔCt_control_ and gene expression is expressed as ^2−ΔΔ^Ct.

### Statistical analysis

Statistical analyses were performed using the SPSS 19 statistical software program (SPSS Inc., Chicago, IL, USA). Analysis of variance was used to analyze the difference between groups, and Tukey’s least significant difference test was used to detect difference between two groups. P<0.05 was considered to indicate a statistically significant difference.

## Results

### Identification of the NP cells

**Figure 1:**
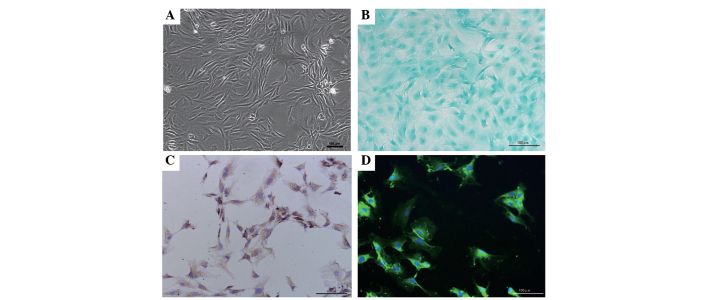
Identification of NP cells. (A) Morphology of NP cells under phase contrast microscopy. (B) Expression of proteoglycan in NP cells, analyzed by Alcian blue staining. (C) Expression of collagen II in NP cells, detected by the immunocytochemistry, under light microscopy, scale bar=100 *µ*m. (D) Expression of cytokeratin 19 in NP cells, detected by the immunofluorescence, under fluorescence microscopy,. Scale bars=100 *µ*m. NP cells, nucleus pulposus.

In order to ensure the purity of the NP, the gelatinous tissue was only harvested when they were extruding out of the endplate following endplate rupture. Under phase-contrast microscopy, the NP cells exhibited an appearance similar to chondrocytes, with polygonal and stellate morphology, and contained vacuoles in the cytoplasm with long processes (Fig. 1A). The expression of proteoglycans and collagen II were detected within the cytoplasm using Alcian blue staining and immunocytochemistry, and this was predominantly distributed around the nucleus (Fig. 1B and C). In addition, CA125, a novel maker gene for NP cells, was also detected in the cells under florescence microscopy, further validating the purity of the cells (Fig. 1D).

### Leptin enhances the expression of catabolic enzymes

**Figure 2:**
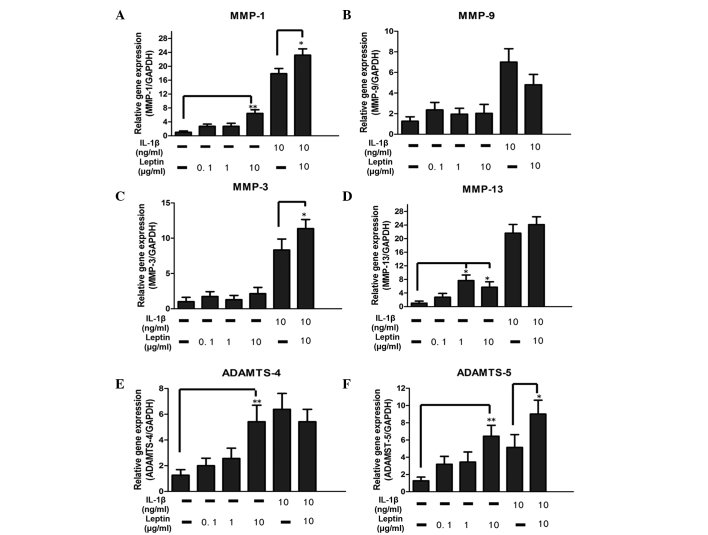
Effect of the leptin, alone or in combination with IL-1β, on catabolic enzymes in NP cells. The NP cells were treated with leptin (0.1, 1 ot 10 *µ*g/ml) either alone or in combination with IL-1β (10 ng/ml) for 48 h. mRNA levels of (A–D) metalloproteases and (E–F) aggrecanases, which were normalized by the expression of GAPDH separately. Data were analyzed using reverse transcription-quantitative polymerase chain reaction. IL-1β was added 30 min prior to the addition of leptin. Data are presented as the mean fold change ± standard deviation (^*^P<0.05 and ^**^P<0.01, compared with the untreated control; n=6). NP cells, nucleus pulposus; IL, interleukin; MMP, matrix metalloproteinase; ADAMTS, a disintegrin and metalloproteinase with thrombospondin motifs; GAPDH, glyceraldehyde phosphate dehydrogenase.

The catabolic enzymes investigated in the present study included MMP-1, MMP-3, MMP-9, MMP-13, ADAMTS-4 and ADAMTS-5. A significant (P<0.05) dose-dependent increase in the mRNA expression levels of *MMP-1*, *MMP-13*, *ADAMTS-4* and *ADAMTS-5*, were observed to be regulated by leptin alone (Fig. 2). Compared with the control group, there was an ~8-fold increase in the mRNA levels of *MMP-1* and *MMP-13* in the leptin-treated group. Although the mRNA expression levels of *MMP-3* and *MMP-9* were not altered by leptin, synergy between leptin (10 *µ*g/ml) and IL-1β enhanced the mRNA expression of *MMP-3*, suggesting that the base level of inflammation may be important in regulation (Fig. 2). In addition, the levels of *MMP-1* and *ADAMTS-5* were also elevated by leptin in combination with 10 *µ*g/ml IL-1β.

### Leptin reduces the mRNA and protein expression levels of collagen II

**Figure 3:**
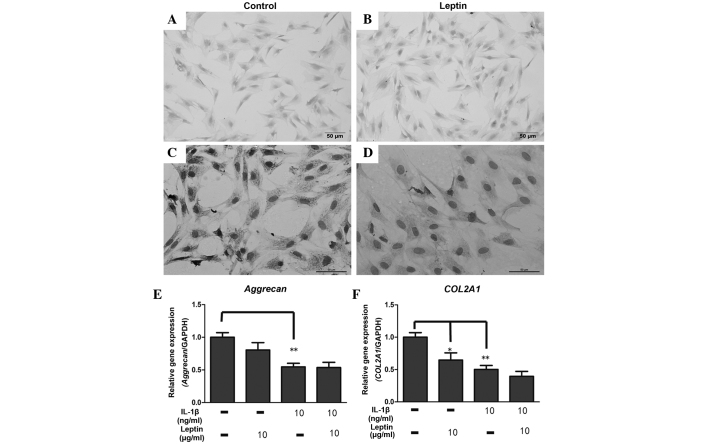
Effect of leptin on the mRNA and protein expression levels of proteoglycan and collagen-II. TheNP cells were incubated with leptin (10 *µ*g/ml) in 10% fetal bovine serum/Dulbecco’s modified Eagle’s medium for 1 week, following which these cells were analyzed using (A and B) Alcian blue staining for proteoglycan and (C and D) immunocytochemistry for collagen II (Scale bar, 50 *µ*m). (E and F) To detect the mRNA level of *aggrecan* and *COL2A1* using reverse transcription-quantitative polymerase chain reaction, the cells were treated with leptin (10 *µ*g/ml) alone or in combination with IL-1β (10 ng/ml) for 48 h. IL-1β was added to the culture 30 min prior to the leptin. Data are expressed as the mean±standard deviation (^*^P<0.05 and ^**^P<0.01, compared with the untreated control, n=6.

According to the above results, the present study subsequently investigated the expression levels of proteoglycan and collagen II, which were degraded by these catabolic enzymes. The intensity of the Alcian blue staining, used to detect the proteoglycan levels, was similar between the control group and the leptin group, in which the cells were treated with leptin (10 *µ*g/ml) for 1 week (Fig. 3A and B). By contrast, the expression of collagen II markedly declined in the leptin-induced cells compared with the control group, as evidenced in the immunocytochemical analysis of collagen II (Fig. 3C and D). The results of the RT-qPCR for *aggrecan* were consistent with those of the Alcian blue staining, demonstrating that 10 *µ*g/ml leptin had no effect on the mRNA expression of *aggrecan* following exposure for 48 h (Fig. 3E). However, the mRNA expression of *COL2A1* was significantly (P<0.05) reduced by leptin (Fig. 3E). When the cells were co-cultured with IL-1β and leptin, no synergistic effect on the expression of ECM was observed.

### Leptin activates the MAPK, PI3K/Akt and JAK2/STAT3 pathways

**Figure 4:**
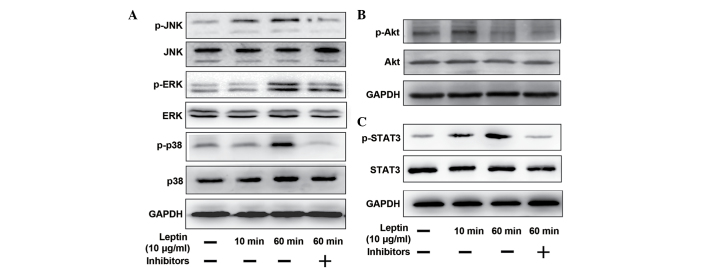
Different signaling pathways regulated by leptin and theit relevant pharmacological inhibitors. The NP cells were treated with leptin (10 *µ*g/ml) alone for 10 min and 60 min, or with leptin (10 *µ*g/ml) in combination with different signaling pathway inhibitors for 60 min, followed by the western blotting. The inhibitors were added 30 min prior to leptin. To regulate the mitogen-activated protein kinase pathways (A) SP600125 (10 *µ*M, JNK inhibitor), U0216 (5 *µ*M, ERK inhibitor) and SB220025 (10 *µ*M, p38 inhibitor) were added. (B) Wortannim (20 nM) was added to inhibit the PI3K/Akt pathway. (C) AG490 (40 *µ*M) was used to inhibit the JAK2/STAT3 pathway. JNK, c-Jun-N-terminal kinase, p-, phosphorylated; ERK, extracellular signal-regulated kinase; PI3K, phosphatidylinositol 3-kinase; JAK, Janus kinase; GAPDH, glyceraldehyde phosphate dehydrogenase.

The mechanism by which leptin regulated the catabolic enzyme in NP cells was investigated by western blotting to analyze the expression levels of the MAPK, PI3K/Akt and JAK2/STAT3 pathways. At 60 min post-stimulation with 10 *µ*g/ml leptin, the expression of p-JNK, p-ERK and p38, which comprise the MAPK pathway, were all activated, evidenced by the increased intensity of the protein bands (Fig. 4A). The induction of Akt phosphorylation was detected as early as 10 min post-stimulation (Fig. 4B), and a time-dependent increase in the phosphorylation of STAT3, induced by leptin, was detected at 60 min (Fig. 4C). Whether the JNK, ERK, p38, PI3K/Akt and JAK2/STAT3 pathway inhibitors (SP600125, U0216, SB220025 wortannim and AG490, repectively) inhibited the activation of these pathways was also examined. As shown in Fig. 4, when the cells were co-treated with leptin and the inhibitors, the phosphorylation level of each protein declined significantly.

### Effect of pathway inhibitors on the expression of catabolic enzymes

**Figure 5:**
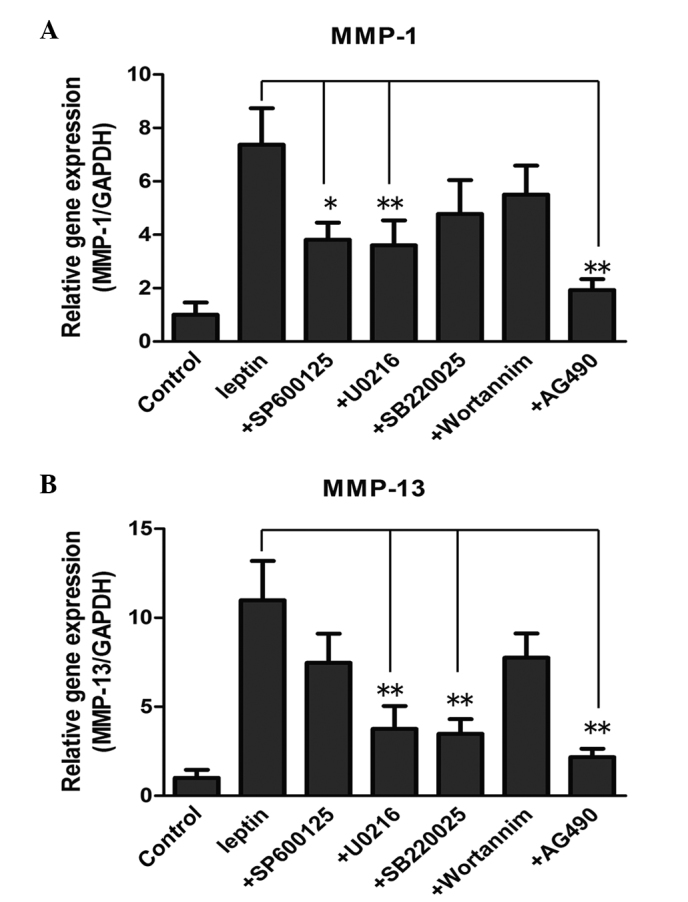
Effect of different pathway inhibitors on the mRNA levels of (A) *MMP-1* and (B) *MMP-13* in NP cells. The NP cells were treated with leptin, alone or in combination with pathway inhibitors, for 48 h. These inhibitors included SP600125 (10 *µ*M, JNK inhibitor), U0216 (5 *µ*M, ERK inhibitor) SB220025 (10 *µ*M, p38 inhibitor), wortannim (20 nM, PI3K/Akt inhibitor) and AG490 (40 *µ*M, JAK2/STAT3 inhibitor), which were added to the culture medium 30 min prior to the addition of leptin. The mRNA levels of *MMP-1* and *MMP-13*, were each normalized against the expression of *GAPDH* and expressed as the mean fold change ± standard deviation compared with the control, detected using reverse transcription-quantitative polymerase chain reaction. ^*^P<0.05 and^**^P<0.01, compared with the control (n=6).

In order to further confirm the involvement of the pathways in the increased expression of enzymes, the NP cells were treated with leptin, either alone or in combination with the pathway inhibitors, followed by RT-qPCR to determine the mRNA levels. With regard to the mRNA level of MMP-1, the JNK inhibitor (10 *µ*M SP600125), ERK inhibitor (5 *µ*M U0216) and JAK2/STAT3 inhibitor (40 *µ*M AG490) reversed the induced by leptin (Fig. 5A; P<0.05). By contrast, the expression levels were not affected by the p38 inhibitor (10 *µ*M SB220025) or the PI3K/Akt inhibitor (20 nM wortannim. The mRNA level of MMP-13 induced by leptin was reduced significantly by SP600125, U0216 and AG490 at the same concentration (Fig. 5B; P<0.05). The PI3K/Akt inhibitor failed to inhibit MMP-1 and MMP-13, suggesting that the PI3K/Akt pathway may not be involved in the process.

## Discussion

The importance obesity in the progression of IDD has attracted significant attention. Previous studies have demonstrated that obesity is a risk factor for LBP, which is associated with IDD (22,23). In addition, compared with that in individuals without IDD, body mass index (BMI), regarded as a measurement of body fat, is significantly higher in southern Chinese patients with IDD (24). In addition, Takatalo *et al* (25) suggested that abdominal diameter (AD), sagittal diameter (SAD) and waist circumference were associated with disc degeneration, according to the magnetic resonance imaging.

The potential mechanisms underlying the effect of obesity on IDD may include increased mechanical loading and atherosclerosis caused by obesity (25), however, the role of adipocytokines, and leptin in particular, in disc degeneration remains to be elucidated. It has been reported that leptin can stimulate the proliferation of rat NP cells and human annulus fibrosus cells *in vitro* (13,14), contributing to the formation of cell clusters, which is a mark of disc degeneration (26). Furthermore, cytoskeleton proteins, including β-actin, F-actin and vimentin, can be dysregulated and reorganized by leptin, indicating cytoskeletal remodeling in leptin-treated cells (21). In the present study, treatment with leptin alone promoted the mRNA expression levels of *MMP-1*, *MMP-13*, *ADAMTS-4* and *ADAMTS-5*, and reduced the protein and mRNA level of collagen II in the NP cells, indicating the pro-catabolic effect of leptin on the metabolism of discs. In addition to the close association between leptin levels and obesity, the results of the present study suggested that leptin may partly account for the effect of obesity on IDD. In addition, the catabolic effect induced by leptin may contribute to the compensatory proliferation of NP cells, however, this requires further investigation.

During the development of IDD, a significant increase in the expression and activity levels of MMPs and AMAMTSs, including MMP-1, 3, 7, 9, and 13, and ADAMTS-1, 4, 5, 9 and 15, have been found (5). The findings of Le Maitre *et al* (27), that MMP-1 and MMP-13 were expressed highly in degenerated NP tissue, may explain why the mRNA expression levels of *MMP-1* and *MMP-13*, rather than *MMP-3* and *MMP-9* were increased significantly by the leptin in the present study. As the predominant target of MMP-13 is collagen II, the mRNA expression of *COL2A1* and protein expression of collagen II were inhibited in the leptin-treated cells. Similarly, Hui *et al* (16) found that leptin significantly induced collagen release in bovine cartilage. Although the mRNA expression of *ADAMTS* in NP cells was increased to a certain extent, the Alcian blue staining and mRNA level of *aggrecan* were similar between the leptin-treated cells and the control cells. This result indicated that the aggrecanase, MMP-3, which was not affected by leptin, was also important in regulating the expression of aggrecan.

The network comprising leptin and pro-inflammatory mediators is complex. In the present study, synergy between leptin and IL-1β was observed in the increased expression levles of MMP-1, MMP-3 and ADAMTS-5, suggesting that leptin sensitized the NP cells to the IL-1β-induced catabolic response *in vitro*. However, the synergy was not observed in the mRNA expression of *COL2A1* and *aggrecan*. It has also been reported that leptin can induce pro-inflammatory mediators, including the IL-6, IL-8, NO and prostaglandin E2 (PGE2), which activate the expression of catabolic enzymes (17,18,28).

Leptin exerts its biological effect in cells via several pathways. JAK2, PI3K, MEK-1 and p38 kinase are all involved in the process and leptin, in synergy with IL-1, enhances the expression of NOS in human chondrocytes (18). In the microglia, the IRS-1/PI3K/Akt/NF-κB and p300 signaling pathway also mediates the production of IL-6, induced by leptin (28). In addition, the increased expression levels of MMP-1, MMP-3 and MMP-13 in chondrocytes, induced by leptin, is associated with the activation of the STAT, MAPK, NF-κB and Akt signaling pathways (15,16). In the present study, the results of the western blotting demonstrated that the MAPK, Akt and JAK2/STAT3 pathways were all activated by leptin in the NP cells, and the Akt pathway was activated earlier than the other pathways. However, when these pathways were inhibited, the p38 and PI3K/Akt pathway were found not to be involved in the regulation of the mRNA expression of *MMP-1*, and the JNK and PI3K/Akt pathways did not affects the regulation of the mRNA expression of *MMP-13*. Neither the mRNA expression of *MMP-1* nor *MMP-13* were affected by the PI3K/Akt pathway, indicating that the PI3K/Akt pathway was not involved in the increase of catabolic enzymes regulated by leptin in NP cells.

In conclusion, the results of the present study suggested that leptin, either alone or in synergy with IL-1β promoted the mRNA expression levels of *MMP-1, MMP-9, MMP-3, ADAMTS-4* and *ADAMTS-5*. In addition, the mRNA level of *COL2A1* and collagen protein declined in the leptin-treated NP cells. Although leptin activated the MAPK, PI3K/Akt and JAK2/STAT3 pathways, the PI3K/Akt pathway was not involved in the regulating the expression of MMP-1 and MMP-13.

## Figures and Tables

**Table I:** Primer sequences for ADAMTSs, MMPs, aggrecan, COL2A1 and GAPDH.

Gene	Primer sequence (5′-3′)
*MMP-1*	Forward: GACCTCATGTTCATCTTTAGA
	Reverse: CACCACAATAAGGAATTCGTT
*MMP-3*	Forward: TGGACCAGGGACCAATGGA
	Reverse: GGCCAAGTTCATGAGCAGCA
*MMP-9*	Forward: GTCCAGACCAAGGGTACAG
	Reverse: GTCCAGACCAAGGGTACAG
*MMP-13*	Forward: GCCGGAAATAACCTCACTGT
	Reverse: CTCACCCTCTACACCTCCCT
*ADAMTS-4*	Forward: GCCGGAAATAACCTCACTGT
	Reverse: CTCACCCTCTACACCTCCCT
*ADAMTS-5*	Forward: CGACAAGAGTCTGGAGGTGAG
	Reverse: CGTGAGCCACAGTGAAAGC
*Aggrecan*	Forward: AGGATGGCTTCCACCAGTGC
	Reverse: TGCGTAAAAGACCTCACCCTCC
*COL2A1*	Forward: CTCAAGTCGCTGAACAACC
	Reverse: CTATGTCCACACCAAATTCC
*GAPDH*	Forward: CCACAGTCCATGCCATCAC
	Reverse: TCCACCACCCTGTTGCTGTA

GAPDH, glyceraldehyde phosphate dehydrogenase; MMP, matrix metalloproteinase; ADAMTS, a disintegrin and metalloproteinase with thrombospondin motifs.
